# Chronic noise exposure exacerbates AD-like neuropathology in SAMP8 mice in relation to Wnt signaling in the PFC and hippocampus

**DOI:** 10.1038/s41598-018-32948-4

**Published:** 2018-10-02

**Authors:** Donghong Su, Wenlong Li, Xiaojun She, Xuewei Chen, Qingfeng Zhai, Bo Cui, Rui Wang

**Affiliations:** 1 School of Medicine and Life Sciences, University of Jinan-Shandong Academy of Medical Sciences, Jinan, China; 2 Department of Operational Medicine, Tianjin Institute of Environmental and Operational Medicine, Tianjin, China; 3 Shandong Academy of Occupational Health and Occupational Medicine, Shandong Academy of Medical Sciences, Jinan, China; 4 School of Public Health and Management, Weifang Medical University, Weifang, China

**Keywords:** SAMP8 Mice, Chronic Noise Exposure, Senescence-accelerated Mouse Prone 8 (SAMP8), Hippocampus, Cornu Ammonis, Alzheimer's disease, Risk factors

## Abstract

Non-genetic environmental hazards are thought to be associated with genetic susceptibility factors that increase Alzheimer’s disease (AD) pathogenesis. Aging and chronic noise exposure have been considered important factors in the AD. Here, we investigated the impact of chronic noise exposure on the AD-like neuropathology in the senescence-accelerated prone mouse (SAMP8) and the underlying mechanisms of such effects. We examined the consequences of AD-like neuropathology in 3-month-old SAMP8 mice using low- and high-intensity noise exposure and 8-month-old SAMP8 mice as aging positive controls. Immunoblotting and immunohistochemistry were conducted to examine AD-like pathological changes and potential mechanisms. Chronic noise exposure led to progressive overproduction of Aβ and increased the hyperphosphorylation of tau at Ser396, Thr205, and Thr231 sites in the hippocampus and the prefrontal cortex (PFC) in young SAMP8 mice, similar to that observed in aging SAMP8 mice. Both noise exposure and aging could cause a significant downregulation in Wnt signaling expression. These findings demonstrate that chronic noise stress exacerbated AD-like neuropathology, possibly by disrupting Wnt signaling and triggering aberrant tau hyperphosphorylation and Aβ in the PFC and hippocampus.

## Introduction

Alzheimer’s disease (AD) is a neurodegenerative disease in which the significant pathological changes are an overproduction of amyloid beta (Aβ) and increased hyperphosphorylation of tau^1^. The complex etiology and pathogenesis of AD explain in part why there is no effective treatment for the disease. It is generally believed that the association between environmental stress, the aging process, and their causal role plays a role in the development of AD pathogenesis^2–4^.

Aging is the major risk factor for AD^5^. The incidence of dementia increases exponentially with increasing age^6^. For all studies combined the incidence of dementia doubles with every 6.3-year increase in age, from 3.9 per 1000 person-years at age 60–64 to 104.8 per 1000 person-years at age 90+^7^. AD-like pathological changes such as aggregation of Aβ and the phosphorylation of tau protein were thought to be associated with aging^8,9^.

Among environmental hazards, noise has always been an important environmental concern for humans. Studies have shown that chronic noise exposure can cause physiological or even pathological changes in the classical auditory system and in non-lemniscal brain regions, such as the hippocampus and cortex^10,11^. Chronic noise exposure in experimental animals can cause significant and persistent hyperphosphorylation of tau and formation of prominent pathological neurofibrillary tangles (NFT) of tau in the hippocampus and the prefrontal cortex **(**PFC), key structures in learning and memory and the initial sites of tau pathology in AD^12^. These results indicate that noise exposure is associated with an increased risk of developing AD.

The development of AD pathogenesis is multifactorial, with aging, environmental and occupational factors implicated^13^. Based on these findings, we theorized that chronic noise exposure would exacerbate aging-related AD-like neuropathology and play important roles in the development of AD, we investigate the effects of chronic noise exposure on AD-like pathology in the senescence-accelerated mouse prone 8 (SAMP8), an excellent model of the accelerated senescence associated with AD. To understand the underlying mechanism, we further evaluated the involvement of Wnt signaling in this process.

## Methods

### Animal use and experimental grouping

The SAMP8 mice used in our study were kindly provided by Tianjin University of Traditional Chinese Medicine. The mice were kept under standard housing conditions with controlled ambient temperature (23 °C ± 2 °C) and humidity (50%–60%) and a 12-h light/12-h dark cycle (lights on from 06:00 to 18:00). They had free access to food and water in their home cages and were allowed to adapt to the laboratory environment for 5 days prior to the start of the experiment.

The 3-month-old male SAMP8 mice were randomly separated into three groups: control group, the low intensity noise exposure group (LN group) that was exposed to 88 dB sound pressure level (SPL) white noise, the high intensity noise exposure group (HN group) exposed to 98 dB SPL white noise, and the control aging group that consisted of 10 male SAMP8 mice at 8 months of age. The LN and HN groups were exposed 4 h/day for 30 days from 8:00 to 12:00, whereas control mice were housed in similar cages but exposed to background noise (<40 dB SPL) from another chamber. After exposure to noise for 30 consecutive days, mice were sacrificed and brain samples were immediately collected for biochemical analyses and stored at –80 °C until use. All experiments were adhered to the guidelines of the National Institute of Health for the use of the experimental animals and were performed in accordance with approved guidelines specified by the Animal and Human Use in Research Committee of the Tianjin Institute of Environmental Medicine and Occupational Medicine.

### Noise exposure set-up

All noise exposures were performed as described in the previous study^14^. Briefly, the noise was generated using a noise generator and amplified with a power amplifier and delivered through a loudspeaker. A B & K 3560 C-size front end and 4938 1/4” pressure-field microphone were used to record the exposure conditions (Brüel and Kjaer Instruments, Denmark). The main spectrum of the noise emitted from the speaker had a range of 400–6300 Hz (1/3 octave bands). Animals were exposed to noise in a reverberation chamber where wire-mesh cages were placed at the center of the sound field. A loudspeaker was suspended directly above the cages. The noise level variation was less than 2 dB within the space available to the animal. The background noise level in the chamber was below 40 dB SPL.

### Determination of protein concentration by ELISA

Frozen mice hippocampi were homogenized in ice-cold 1 x phosphate buffered saline (0.02 mol/L, pH 7.0–7.2). Total protein concentrations were determined by the bicinchoninic acid method (Boster, Wuhan, China). Protein levels for Aβ 1–40 and Aβ 1–42 were determined in hippocampal homogenates using ELISA kits (BlueGene Biotech, Shanghai, China), according to the manufacturer’s recommendations (www.bluegene.cc). Concentration values of Aβ 1–40 and Aβ 1–42 were normalized to total hippocampal protein levels. All concentrations were expressed in nanograms per gram of total protein and were defined as the average of duplicates for given mice.

### Detection of Aβ by thioflavin T staining

After cardiac perfusion with 4% paraformaldehyde, brain tissues were post-fixed in 4% paraformaldehyde for 72 h, paraffin embedded, and sectioned at 5 μm. The paraffin sections were placed in GENMED reagent A for 45 min then GENMED reagent B for 9 min. Slices were placed in a dye solution (GMS 80100.1, GenMed Scientifics Lnc, USA) for 10 min then subsequently washed. Sections were coverslipped with Antifade Mounting Medium (Cat No.S2110, Solarbio) and imaged on a fluorescence microscope (Olympus DP71, Japan).

### Western Blot analysis

The hippocampus preparation and Western Blot was performed as described previously^15^. Briefly, the tissue was dissected and homogenized in ice-cold radioimmunoprecipitation assay buffer (Cat No.R0020, Solarbio). Homogenates were spun at 14,000 × g for 15 min at 4 °C, and the supernatants were collected. Later, 10 μg/lane of the sample was run on 10% sodium dodecyl sulfate-polyacrylamide gels (Cat No. M00657, GenScript, USA) and transferred to microporous polyvinylidene fluoride membranes (0.45 μm, F. Hoffmann-La Roche Ltd., Germany). Immunoblots were probed with specific antibodies, as described in section 4.5, and incubated with peroxidase-conjugated species-specific anti-IgG secondary antibodies. After visualizing by enhanced chemiluminescence (EMD Millipore Co., USA), the intensity values of the immunoreactive signals were quantified by using Gel-Pro 3.1 software (Media Cybernetics Inc., USA).

### Primary antibodies

Aβ (polyclonal, 1:200; Santa Cruz Biotechnology, USA), T-tau (polyclonal, 1:1000; Santa Cruz Biotechnology, USA), PS205 (polyclonal, 1:1000; Bioworld Technology, China), PS396 (polyclonal, 1:1000; Santa Cruz Biotechnology, USA), and PS231 (polyclonal, 1:1000; Bioworld Technology, China) are specific to tau phosphorylated at the residues indicated. GSK-3β (polyclonal, 1:1000; Bioworld Technology, China), GSK-3β Ser^9^ (polyclonal, 1:1000; Bioworld Technology, China), Dvl (polyclonal, 1:100; Santa Cruz Biotechnology, USA), β-catenin (polyclonal, 1:1000; Boster Technology, USA), β-catenin (S33/37, polyclonal, 1:1000; Boster Technology, USA), Dkk-1 (polyclonal, 1:100; Bioworld Technology), and p53 (polyclonal, 1:100; Santa Cruz Biotechnology, USA) antibodies were used to detect endogenous levels of Wnt. Anti-GAPDH (1:10,000; Bioworld Technology) was used as the internal reference standard. Rabbit anti-p-tau (T231) (polyclonal, 1:25; Bioworld Technology) was used to label p-tau in the immunofluorescence assays.

### Immunofluorescence microscopy

Paraformaldehyde-fixed paraffin embedded 5 μm sections were washed in TBS, incubated for 30 min in 0.5% Triton X-100 to permeabilize the tissue. Sections were blocked with 10% goat serum(Cat No.ZLI-9021, ZSGB-BIO) in TBS for 2 h at 37 °C to reduce nonspecific binding then incubated for 1 h at 37 °C with anti-p-tau (T231) diluted in TBST with 10% goat serum before incubating overnight at 4 °C. The following day, the sections were washed and incubated with the FITC-labeled anti-rabbit antibody (1:25, ZSGB-BIO), diluted in TBS with 10% goat serum, for 1 h at 37 °C. After washing, the sections were coverslipped with Antifade Mounting Medium and imaged on a fluorescence microscope.

### Statistics

Data were analyzed using SPSS v.19.0 software (SPSS Inc., Chicago, IL, USA). To determine statistical significance in noise exposure group with the control group, we performed a one-way analysis of variance followed by an LSD-test where P < 0.05 was considered statistically significant. Then, Student’s t-test was performed to determine statistical significance compared with the aging group where P < 0.05 was considered statistically significant. The data presented in the graphs indicate group means ± standard deviation.

## Results

### Chronic noise-induced aggregation of Aβ in the hippocampus and PFC

**Figure 1:**
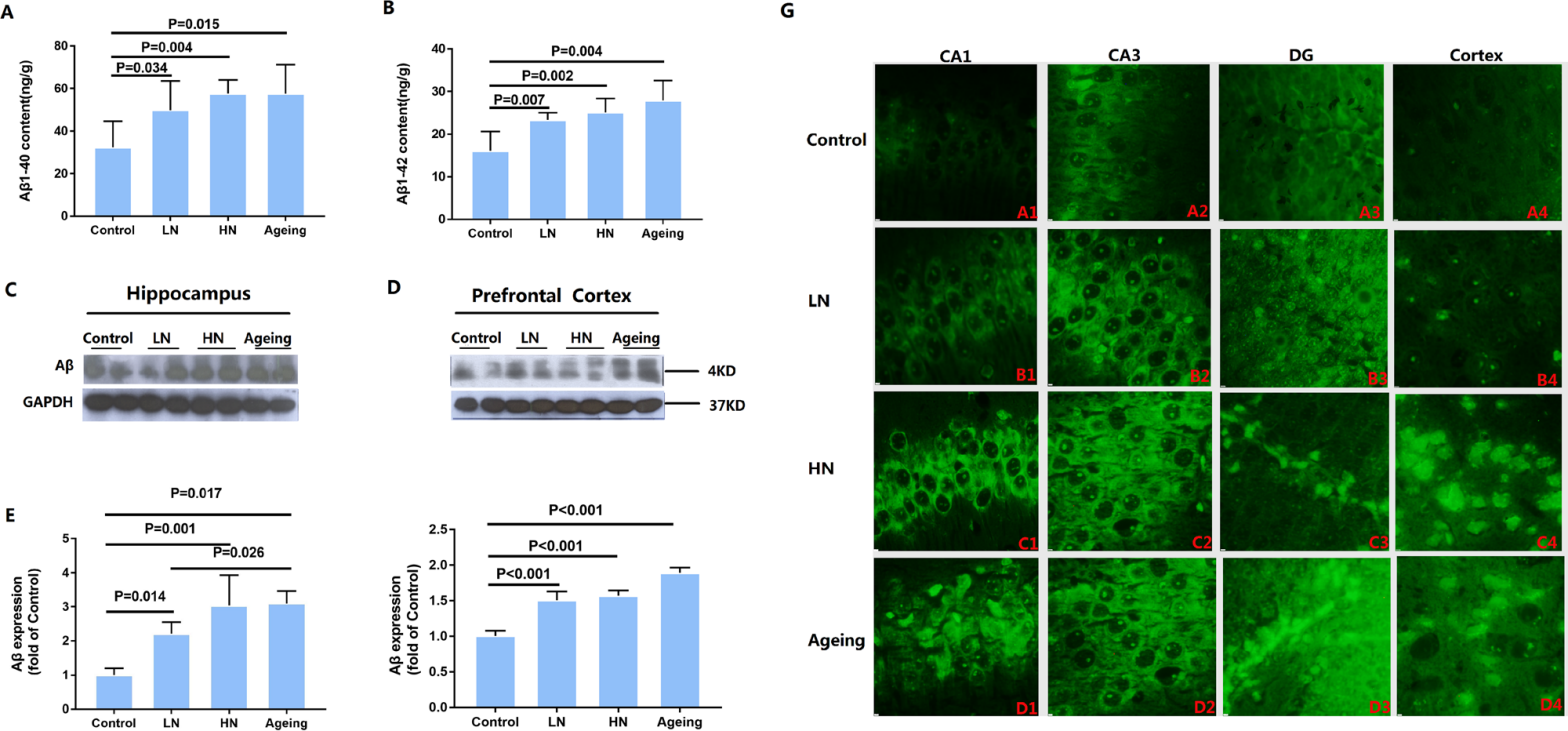
Chronic noise-induced changes of Aβ in SAMP8 mice. Quantification of Aβ 1–40 and Aβ 1–42 level by ELISA (**A**,**B**). Western Blot analysis of Aβ expression in the hippocampus (**C**) and PFC (**D**) in SAMP8 mice. Quantification of immunoreactive band density measured in Panels C and D, normalized against GAPDH. Quantification of immunoreactive band density measured in E and F. Data are represented as a percent change relative to the control (n = 6 per group). Data are shown as the mean ± standard deviation. HN, high-intensity noise exposure; LN, low-intensity noise exposure. Results were normalized as the control = 100%. The result of the distribution patterns of Aβ by thioflavin T (**G**). Representative images of hippocampal CA1 (A1, B1, C1, D1), CA3 (A2, B2, C2, D2), DG (A3, B3, C3, D3), and PFC (A4, B4, C4, D4) immediately after cessation of noise exposure. Scale bar = 15 μm. CA,Cornu Ammonis; DG, Dentate Gyrus; PFC, prefrontal cortex.

In order to evaluate the effect of chronic noise exposure on the production of Aβ, we performed ELISA and immunoblotting assays to determine the relative levels of Aβ 1–40, Aβ 1–42 and Aβ in hippocampus tissue. The amount of Aβ 1–40 (F_(3,19)_ = 5.016, *P* = 0.012) was significantly higher both in noise exposure group and aging group (Fig. 1A). And the assessment of the Aβ 1–42 (F _(3, 19)_ = 9.007, *P* = 0.001) content revealed a similar trend (Fig. 1B). Expression levels of Aβ were analyzed by quantitative immunoblot analysis in hippocampal and PFC extracts in RIPA buffer from individual mice sacrificed after the end of noise exposure (Fig. 1C and D). Expression of Aβ both in hippocampal (F _(2, 11)_ = 13.053, *P* = 0.002) and PFC (F _(2, 17)_ = 67.741, *P* < 0.001) significantly increased after exposure to both 88 dB and 98 dB white noise for 30 days, with a more significant trend in the high-intensity noise exposure (HN) group which was close to the levels found in the aging group (Fig. 1E and F). This indicates that chronic noise exposure accelerated senescence-related AD-like pathological alterations.

To determine the distribution patterns of Aβ in the different regions of the hippocampus and PFC, we analyzed brain paraffin sections stained with thioflavin T. Expression of Aβ was observed in Cornu Ammonis (CA) 1, Cornu Ammonis (CA) 3, and Dentate Gyrus (DG), but at low levels in the hippocampus and PFC of control mice (Fig. 1G). To a certain extent, the result of the distribution patterns of Aβ by thioflavin T well-reflected the results of our immunoblot.

### Chronic noise-induced tau phosphorylation in the hippocampus and PFC

**Figure 2:**
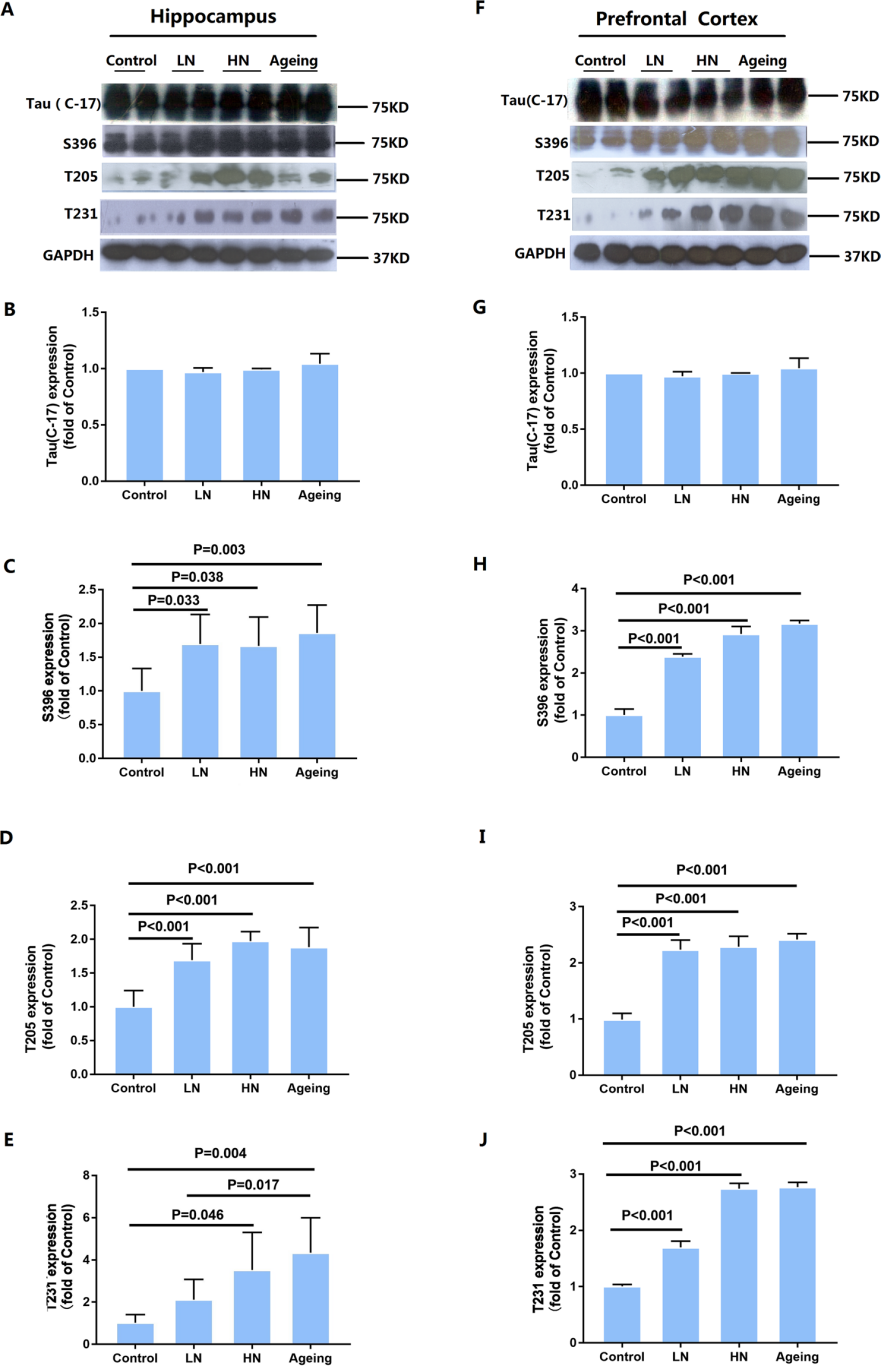
Chronic noise exposure increases hyperphosphorylated tau in the hippocampus (**A**) and PFC (**F**) in SAMP8 mice. Quantification of immunoreactive band density measured in Panels A and F, normalized against GAPDH. The density of the immunoreactive bands was quantified and represented as a percent change relative to the control (**B**–**E**, **G**–**J**). Data are shown as the mean ± standard deviation (n = 6 per group). Results were normalized as the control = 100%.

To assess the influence of chronic noise exposure on the phosphorylation of tau, western blot analysis was used to detect T-tau and phosphorylated tau (p-tau) at Ser396, Thr205, and Thr231 sites in hippocampus and PFC extracts (Fig. 2A and F). Similar expression patterns of T-tau and p-tau were also observed in the hippocampus and PFC of noise-exposed and aging groups, with significantly increased levels at the Ser396 (hippocampus, F_(2,17)_ = 5.793, *P* = 0.014; PFC, F_(2, 17)_ = 322.952, *P* < 0.001), Thr205 (hippocampus, F_(2, 17)_ = 33.396, *P* < 0.001; PFC, F_(2,17)_ = 129.111, *P* < 0.001), and Thr231(hippocampus, F_(2,17)_ = 8.131, *P* = 0.001; PFC, F_(2,17)_ = 620.429, *P* < 0.001) sites (Fig. 2B–E,G–J). These findings indicate that chronic noise exposure aggravated tau phosphorylation in SAMP8 mice.

**Figure 3:**
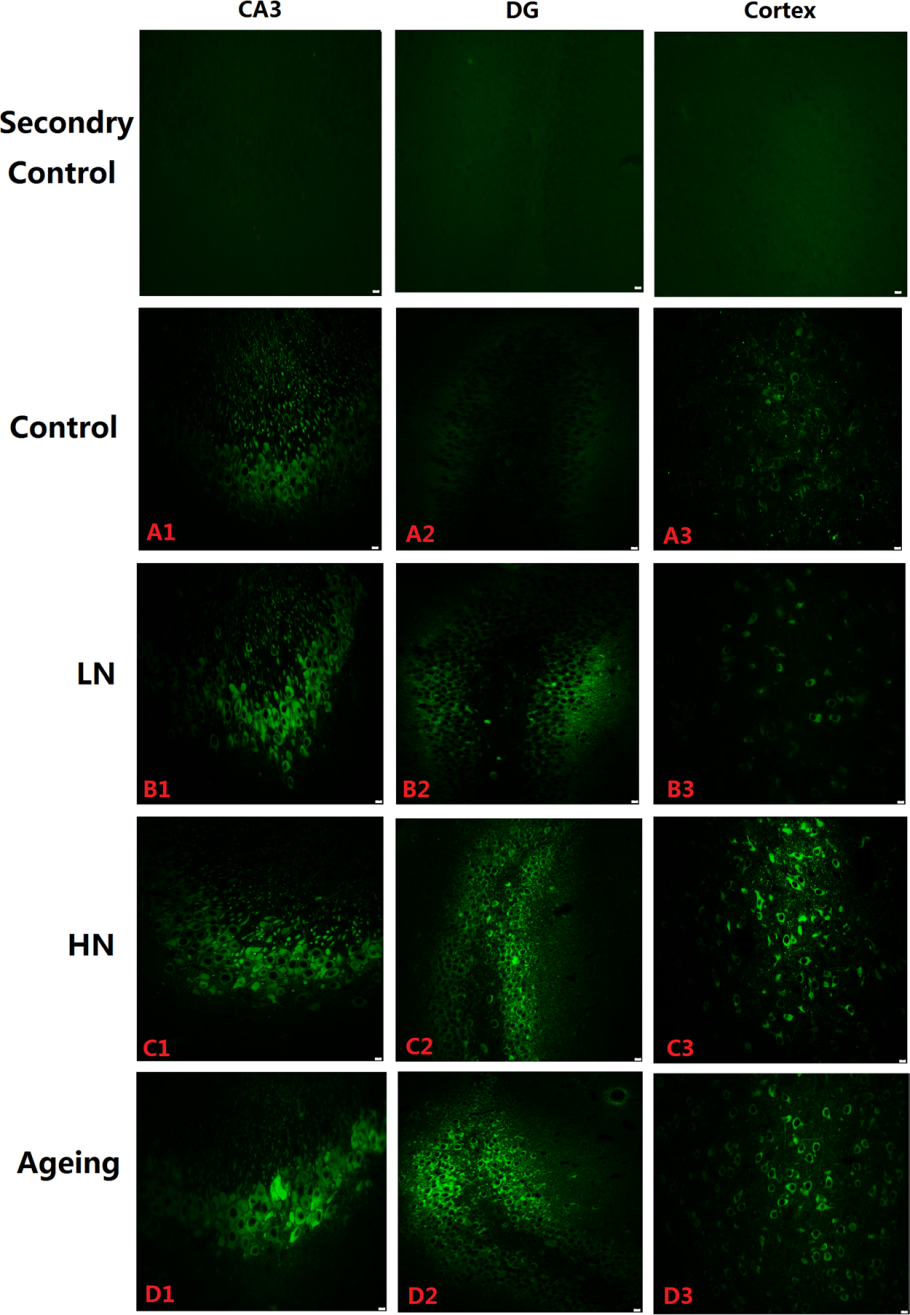
Immunofluorescence staining. P-tau (PT231) expression in the CA3 (A1, B1, C1, D1) and DG (A2, B2, C2, D2) regions of the hippocampus and PFC (A3, B3, C3, D3) in SAMP8 mice. Scale bar = 10 μm. PFC, prefrontal cortex.

To determine the distribution patterns of p-tau in the hippocampus and the prefrontal cortex, we analyzed brain paraffin sections by immunofluorescence. P-tau (Thr231) immunoreactivity was observed in the CA3, DG, and cortex both in noise exposure group and aging group, but at low levels in the control group hippocampi (Fig. 3A). In noise-exposed mice, however, p-tau immunoreactivity was substantially higher in CA3 and DG pyramidal cell layers, and in the cortex cell layer compared to the control group (Fig. 3B and C). Furthermore, we found the same results in the aging group (Fig. 3D). Thus, our immunohistochemical staining results mirrored our immunoblot results.

### Chronic noise-induced inhibition of Wnt signaling in the hippocampus and PFC

**Figure 4:**
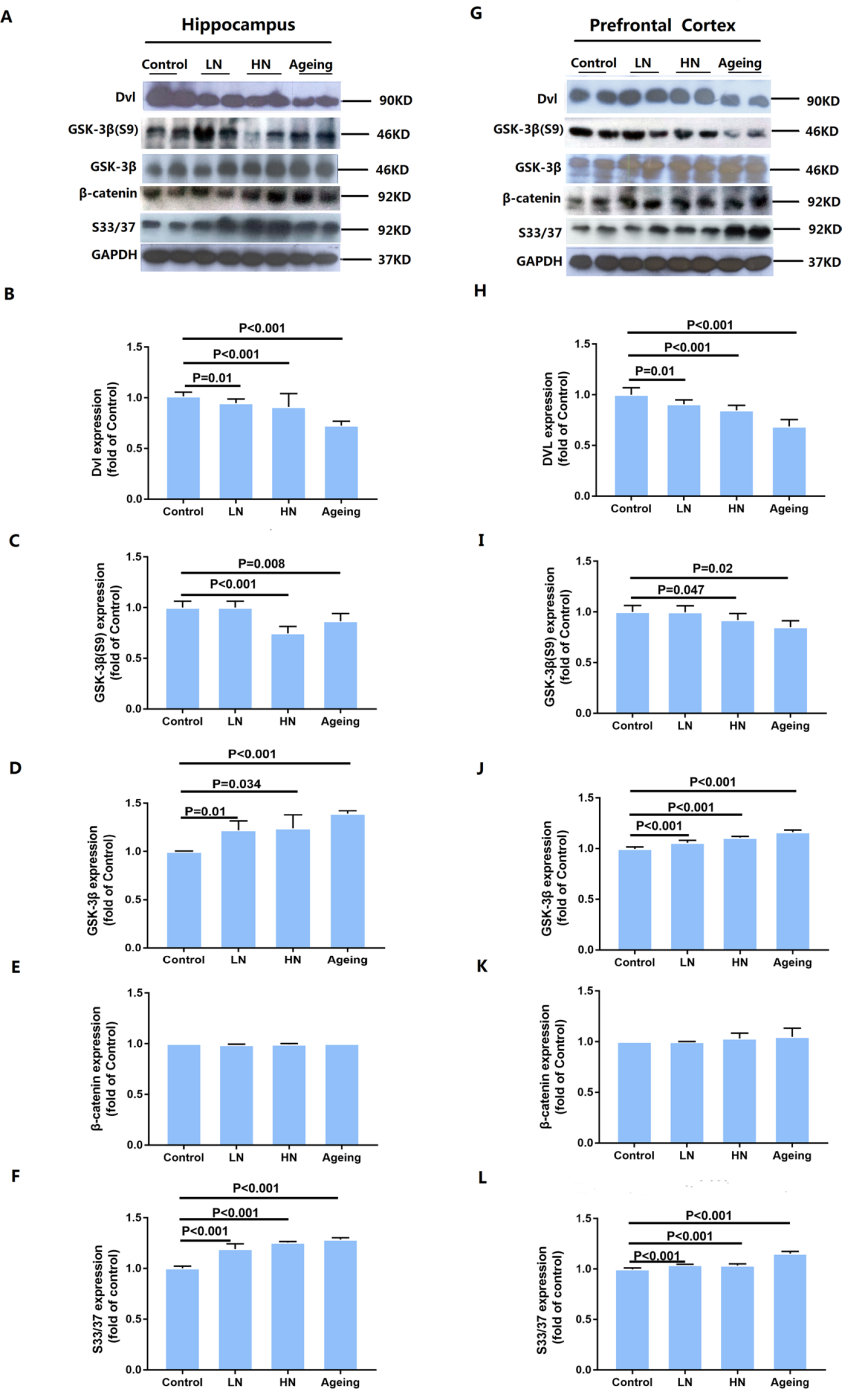
Proteins expression levels of Wnt signaling in the hippocampus (**A**) and PFC (**G**) in SAMP8 mice. Quantification of immunoreactive band density measured in Panels A and G, normalized against GAPDH. The density of the immunoreactive bands was quantified and presented as a percent change relative to the control (**B**–**F**, **H**–**L**). Data are shown as the mean ± standard deviation (n = 6 per group). Results were normalized as the control = 100%. PFC, prefrontal cortex.

To investigate the inhibition of the Wnt pathway, we evaluated the protein levels of glycogen synthase kinase 3β (GSK-3β), GSK-3β Ser^9^, disheveled (Dvl), and β-catenin in the hippocampus and PFC (Fig. 4A and G) by Western Blot. We first detected expression of Dvl protein, which promotes the transcription of the Wnt signal. The results showed that Dvl (hippocampus, F _(2, 17)_ = 43.908, *P* = 0.118; PFC, F _(2, 17)_ = 11.976, *P* = 0.001) protein expression decreased after noise exposure, which is the same as the aging group (Fig. 4B and H). And the expression of GSK-3β Ser^9^ (hippocampus, F _(2, 17)_ = 30.496, *P* < 0.001; PFC, F _(2, 17)_ = 3.005, *P* = 0.08) was decreased after noise exposure (Fig. 4C and I). Interestingly, we found that GSK-3β (hippocampus, F _(2, 17)_ = 11.604, *P* = 0.001; PFC, F _(2, 17)_ = 51.197, *P* < 0.001) increased significantly in the noise exposure group while the aging group to a degree consistent with that of the noise group (Fig. 4D and J). As a negative modulator of Wnt signaling, GSK-3β can induce β-catenin phosphorylation at Ser33, Ser37, and Thr41. The results confirmed that the level of total β-catenin shown no significant change but phosphorylated at Ser33 and Ser37 (hippocampus, F _(2, 17)_ = 95.873, *P* < 0.001; PFC, F _(2, 17)_ = 22.153, *P* < 0.001) sites were not only present in the noise-exposure group but also in the aging group (Fig. 4E,F,K and L).

**Figure 5:**
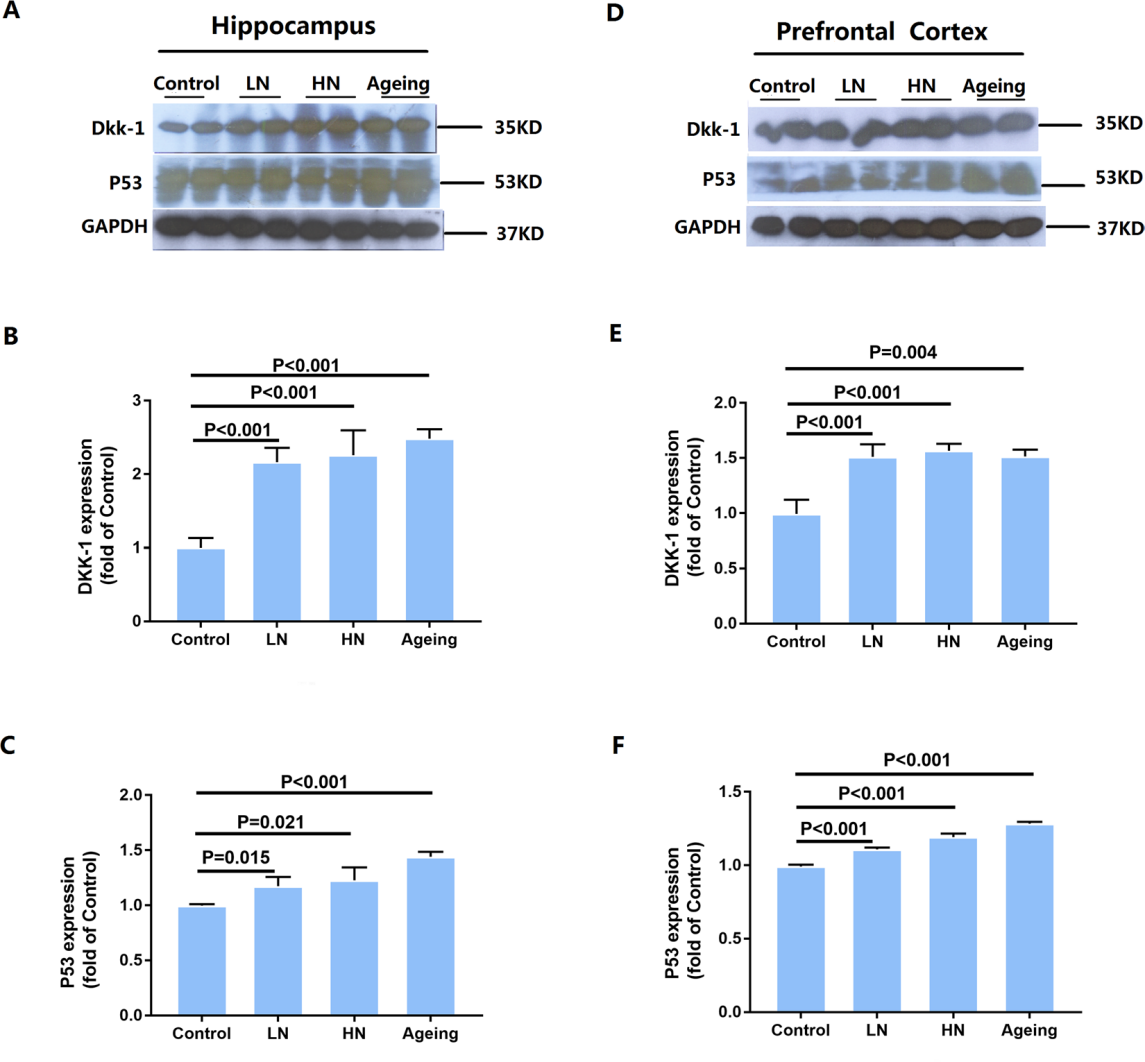
Chronic noise exposure increases the expression of Dkk-1 and p53 in the hippocampus and PFC. Quantification of immunoreactive band density measured in A and D were normalized against GAPDH. Data are represented as a percent change relative to the control (**B**,**C**,**E** and **F**). Bars represent means ± SD, compared to the controls (n = 6 per group). Results were normalized as the control = 100%. PFC, prefrontal cortex.

**Figure 6:**
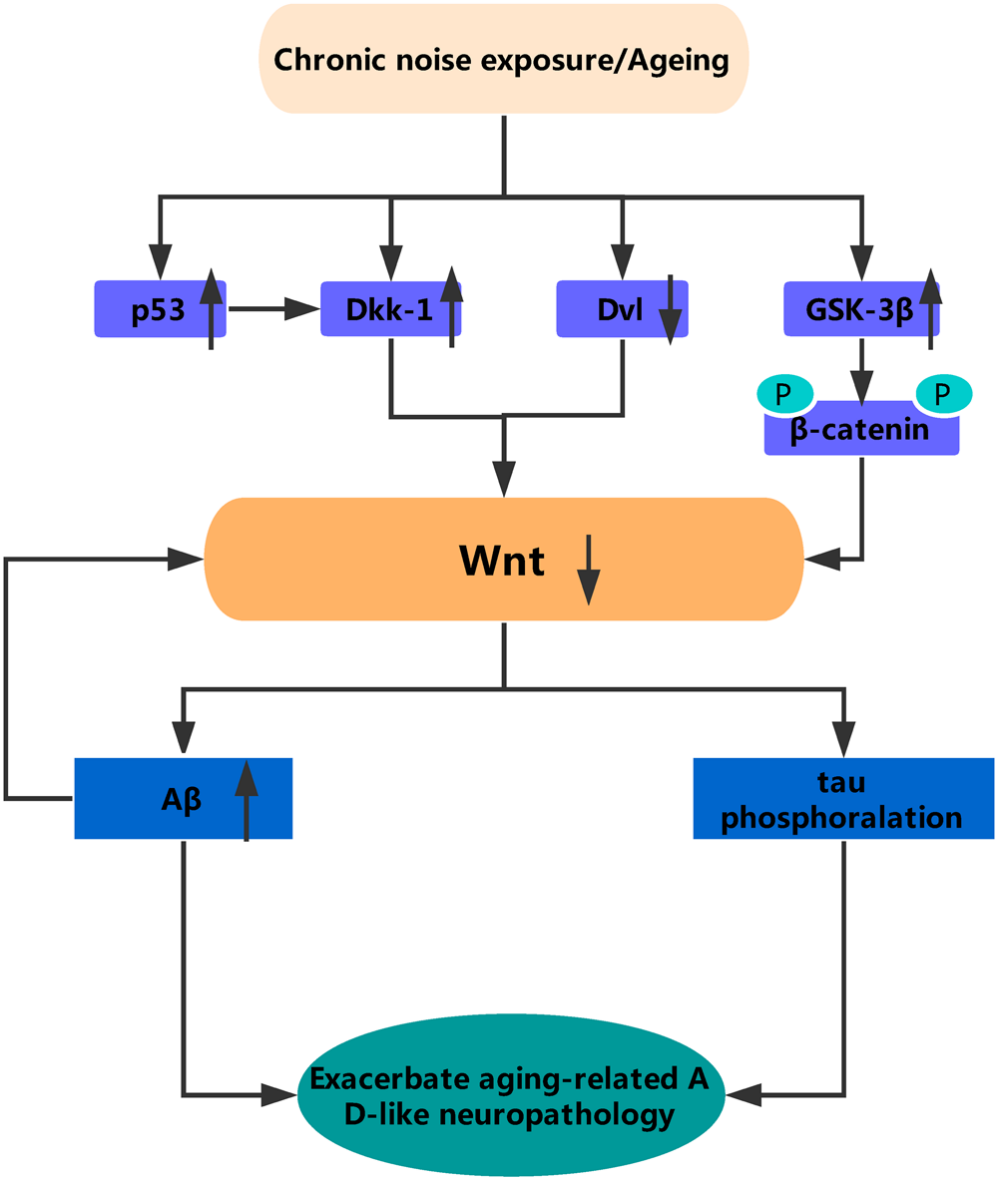
A simplified model of the relationship between Wnt signaling and AD pathology. Chronic noise exposure and aging could induce activation of Dkk-1, a Wnt pathway inhibitor. In the one hand, in absence of Wnt ligands, Dvl protein expression decreased and both β-catenin and tau are phosphorylated by GSK-3β, in the other hand, without the inhibition of Wnt, the expression of Aβ significantly increased, which further exacerbates the downregulation of wnt. All of this indicates that the AD-like neuropathology observed in mice of accelerated aging after chronic noise is likely to be the result of dysregulation of Wnt signaling. Dkk-1, Dickkopf-related protein 1; Dvl, disheveled; p, phosphorylation.

Next, we examined the protein levels of Dickkopf-related protein 1 (Dkk-1) and tumor protein 53 (p53). Dkk-1 inhibits Wnt signaling and is induced by p53. Statistical analysis indicated that chronic noise exposure and age significantly correlated with Dkk-1 (hippocampus, F_(2,17)_ = 52.864, *P* < 0.001; PFC, F_(2,17)_ = 53.640, *P* < 0.001) protein levels, manifested by a significant increase in the noise-exposure and aging groups in both the hippocampus and PFC, as well as an increase in p53 levels (hippocampus, F_(2,17)_ = 12.542, *P* = 0.001; PFC, F_(2,17)_ = 204.120, *P* < 0.001) (Fig. 5A–F). Taken together, these results point to a downregulation of Wnt signaling induced by chronic noise exposure and aging in the hippocampus and PFC of SAMP8 mice (Fig. 6). These results suggest that chronic noise exposure is likely to promote the adverse aging effects through the inhibition of Wnt signaling.

## Discussion

Previous studies have demonstrated that acute or chronic noise exposure could induce various indicators of AD-like pathological changes^11,16–19^ that increase the risk of AD development. In this study, we evaluated the effects of chronic noise exposure in AD-like neuropathological alterations in SAMP8 mice. The present study delivers new insights into the link between noise exposure and AD. Our results represent a comprehensive description of the *in vivo* after-effects of noise exposure on both Aβ accumulation and tau phosphorylation in spontaneous mice of accelerated aging. Our study also implicates Wnt signaling in noise-induced augmentation of AD-like pathology in accelerated aging mice.

It was established that chronic noise exposure could induce AD-like pathology. Following noise stress, we detected significantly higher levels of Aβ protein expression in the hippocampi and PFC. According to the amyloid-cascade hypothesis of the AD, production of the more aggregate-prone Aβ42 is considered to be the key event in AD pathology^20,21^. Aβ peptides originate from proteolysis of the β-Amyloid precursor protein (APP) by the sequential enzymatic actions of β-secretase and γ–secretase^22,23^. Our previous results demonstrated that a persistent increase in Aβ(1–40) and Aβ(1–42) peptide levels and the expression of APP and β-/γ-secretase occurs in the hippocampi of rats following chronic noise exposure^19^. Notably, we confirmed the overexpression of Aβ in the hippocampus and cortex of SAMP8 mice through immunoblot and immunofluorescence. When it comes to phosphorylation tau, we found an increase in the phosphorylation level of tau protein at Ser396, Thr205, and Thr231 sites in the hippocampus and PFC extracts in both the HN group and the aging group. Therefore, there is maybe a combined accelerative impact of noise exposure and aging on contributing to the risk of AD onset and progression.

The SAMP8 mouse is an excellent model to study both aging changes and early AD changes in comparison to the transgenic mice^24^. It has been reported that SAMP8 mice present with the cognitive deficit and other AD-like neuropathological alterations from young ages (5–6 months)^25^. Therefore, we selected 3 months SAMP8 mice to investigate the relationship between chronic noise exposure and the progression of the AD. On the other hand, we chose 8 months SAMP8 mice as aging positive control group to study if there is a possibility that chronic noise-exposure would exacerbate age-related AD-like pathology. Our data of present study showed there is a series of the AD-like neuropathological alterations in chronic noise exposed mice, as well as or even worse than that in aging mice. All of these findings indicate that chronic noise may exacerbate age-related AD-like pathology.

The molecular mechanisms of chronic noise exposure and aging on AD-like pathology seem to be complex. Growing evidence has suggested that the Wnt canonical pathway could be involved in the pathogenesis of AD^26–28^. On one hand, in the absence of a Wnt ligand, GSK-3β facilitates β-catenin phosphorylation, which is degraded in the proteasome leading to loss of Wnt/β-catenin signaling function^29^. GSK3β also has been shown to phosphorylate tau and may have a role in the production of paired helical filaments (PHF) in the human brain^30^. The lasting increases in GSK3β in close correspondence with increases in tau hyperphosphorylation observed in this study suggest that GSK3β might play a causal role in the induction and persistence of tau hyperphosphorylation consistent with our previous studies^12,14^. On the other hand, the Wnt pathway is neuroprotective against Aβ toxicity^31^. Active Wnt/β-catenin signaling favors the generation of non-amyloidogenic forms of APP, thereby reducing the production of the Aβ42 peptide and its aggregates^32,33^. It is interesting that the Aβ peptide that has been reported could inhibit Wnt/β-catenin signaling^34^. Otherwise, Aβ neurotoxicity significantly enhances the expression of Dkk-1 as reported in both cultured cortical neurons and brain tissue samples of AD patients^35^. In the present study, we found down-regulation of Wnt signaling in both noise exposure and aging groups. Hence, there may be a synergy between chronic noise exposure and aging in AD by inhibiting Wnt signaling.

## Conclusions

In summary, our results demonstrated that chronic noise can exacerbate AD-like neuropathology in spontaneous mice of accelerated aging, which may contribute to cognitive deficits that are characteristic of neurodegenerative diseases like AD. Additionally, our findings indicate that the accelerated abnormalities in AD-like neuropathology observed in mice of accelerated aging after chronic noise is likely to be the result of dysregulation of Wnt signaling (Fig. 6). These data suggest that genetic and environmental influences could be one mechanism behind the wide variation in the onset and progression of the AD. Further detailed studies are required to clarify the molecular mechanisms underlying the regulation of Wnt signaling in noise-induced neuropathological changes in the AD.

## References

[1] Querfurth HW, LaFerla FM (2010). Alzheimer’s disease. N. Engl. J. Med..

[2] Gandy S (2011). Perspective: prevention is better than cure. Nature..

[3] Sotiropoulos I (2008). Stress and glucocorticoid footprints in the brain-the path from depression to Alzheimer’s disease. Neurosci Biobehav Rev..

[4] Lupien SJ (1999). Increased cortisol levels and impaired cognition in human aging: implication for depression and dementia in later life. Rev Neurosci..

[5] Fjell AM, McEvoy L, Holland D, Dale AM, Walhovd KB (2014). What is normal in normal ageing? Effects of ageing, amyloid and Alzheimer’s disease on the cerebral cortex and the hippocampus. Prog. Neurobiol..

[6] Ma Q (2011). Age-related autophagy alterations in the brain of senescence accelerated mouse prone 8 (SAMP8) mice. Exp. Gerontol..

[7] Realdon O (2016). Technology-enhanced multi-domain at home continuum of care program with respect to usual care for people with cognitive impairment: the Ability-TelerehABILITation study protocol for a randomized controlled trial. BMC Psychiatry..

[8] Wyss-Coray T (2016). Ageing, neurodegeneration and brain rejuvenation. Nature..

[9] Elobeid A, Libard S, Leino M, Popova SN, Alafuzoff I (2016). Altered Proteins in the Ageing Brain. J. Neuropathol. Exp. Neurol..

[10] Manikandan S (2006). Effects of chronic noise stress on spatial memory of rats in relation to neuronal dendritic alteration and free radical-imbalance in hippocampus and medial prefrontal cortex. Neurosci. Lett..

[11] Cui B, Wu M, She X (2009). Effects of chronic noise exposure on spatial learning and memory of rats in relation to neurotransmitters and NMDAR2B alteration in the hippocampus. J Occup Health..

[12] Cui B (2012). Chronic noise exposure causes persistence of tau hyperphosphorylation and formation of NFT tau in the rat hippocampus and prefrontal cortex. Exp. Neurol..

[13] Duthey, B. Background paper 6. 11: Alzheimer disease and other dementias, A Public Health Approach to Innovation, 1–74 (2013).

[14] Li K (2014). Role of NMDA receptors in noise-induced tau hyperphosphorylation in rat hippocampus and prefrontal cortex. J. Neurol. Sci..

[15] Gai Z (2016). Effects of chronic noise on mRNA and protein expression of CRF family molecules and its relationship with p-tau in the rat prefrontal cortex. J. Neurol. Sci..

[16] Zhang LF (2012). Increased hippocampal tau phosphorylation and axonal mitochondrial transport in a mouse model of chronic stress. Int. J. Neuropsychopharmacol..

[17] Naqvi F, Haider S, Batool Z, Perveen T, Haleem DJ (2012). Sub-chronic exposure to noise affects locomotor activity and produces anxiogenic and depressive like behavior in rats. Pharmacol Rep..

[18] Cheng L, Wang SH, Chen QC, Liao XM (2011). Moderate noise induced cognition impairment of mice and its underlying mechanisms. Physiol. Behav..

[19] Cui B (2015). Chronic Noise Exposure Acts Cumulatively to Exacerbate Alzheimer’s Disease-Like Amyloid-β Pathology and Neuroinflammation in the Rat Hippocampus. Sci Rep..

[20] Hardy J (2006). Alzheimer’s disease: the amyloid cascade hypothesis: an update and reappraisal. J. Alzheimers Dis..

[21] Musiek ES, Holtzman DM (2015). Three dimensions of the amyloid hypothesis: time, space and ‘wingmen’. Nat. Neurosci..

[22] Zhang H, Ma Q, Zhang Y, Xu H (2012). Proteolytic processing of Alzheimer’s β-amyloid precursor protein. J. Neurochem..

[23] Chen M (2015). The Maze of APP Processing in Alzheimer’s Disease: Where Did We Go Wrong in Reasoning?. Front Cell Neurosci..

[24] Morley JE, Farr SA, Kumar VB, Armbrecht HJ (2012). The SAMP8 mouse: a model to develop therapeutic interventions for Alzheimer’s disease. Curr. Pharm. Des..

[25] Bayod S (2015). Downregulation of canonical Wnt signaling in hippocampus of SAMP8 mice, Neurobiol. Ageing..

[26] De Ferrari GV (2014). Wnt/β-catenin signaling in Alzheimer’s disease. CNS Neurol Disord Drug Targets..

[27] Jin N (2017). Sodium selenate activated Wnt/β-catenin signaling and repressed amyloid-β formation in a triple transgenic mouse model of Alzheimer’s disease. Exp. Neurol..

[28] Vallée A, Lecarpentier Y (2016). Alzheimer Disease: Crosstalk between the Canonical Wnt/Beta-Catenin Pathway and PPARs Alpha and Gamma. Front Neurosci..

[29] Wang B (2017). Inhibition of GSK-3β Activation Protects SD Rat Retina Against N-Methyl-N-Nitrosourea-Induced Degeneration by Modulating the Wnt/β-Catenin Signaling Pathway. J. Mol. Neurosci..

[30] Hanger DP, Betts JC, Loviny TL, Blackstock WP, Anderton BH (1998). New phosphorylation sites identified in hyperphosphorylated tau (paired helical filament-tau) from Alzheimer’s disease brain using nanoelectrospray mass spectrometry. J. Neurochem..

[31] Lambert C, Cisternas P, Inestrosa NC (2016). Role of Wnt Signaling in Central Nervous System Injury. Mol. Neurobiol..

[32] Lammich S (1999). Constitutive and regulated alpha-secretase cleavage of Alzheimer’s amyloid precursor protein by a disintegrin metalloprotease. Proc. Natl. Acad. Sci. USA.

[33] Postina R (2004). A disintegrin-metalloproteinase prevents amyloid plaque formation and hippocampal defects in an Alzheimer disease mouse model. J. Clin. Invest..

[34] Magdesian MH (2008). Amyloid-beta binds to the extracellular cysteine-rich domain of Frizzled and inhibits Wnt/beta-catenin signaling. J. Biol. Chem..

[35] Caricasole A (2004). Induction of Dickkopf-1, a negative modulator of the Wnt pathway, is associated with neuronal degeneration in Alzheimer’s brain. J. Neurosci..

