# Author Correction: Chronic noise exposure exacerbates AD-like neuropathology in SAMP8 mice in relation to Wnt signaling in the PFC and hippocampus

**DOI:** 10.1038/s41598-022-10910-9

**Published:** 2022-04-26

**Authors:** Donghong Su, Wenlong Li, Xiaojun She, Xuewei Chen, Qingfeng Zhai, Bo Cui, Rui Wang

**Affiliations:** 1 School of Medicine and Life Sciences, University of Jinan-Shandong Academy of Medical Sciences, Jinan, China; 2 Department of Operational Medicine, Tianjin Institute of Environmental and Operational Medicine, Tianjin, China; 3 Shandong Academy of Occupational Health and Occupational Medicine, Shandong Academy of Medical Sciences, Jinan, China; 4 School of Public Health and Management, Weifang Medical University, Weifang, China

Correction to: *Scientific Reports* 10.1038/s41598-018-32948-4, published online 02 October 2018

This Article contains an error in panel G, image C2 of Figure 1. The revised Figure 1 and accompanying legend appear below.

**Figure 1:**
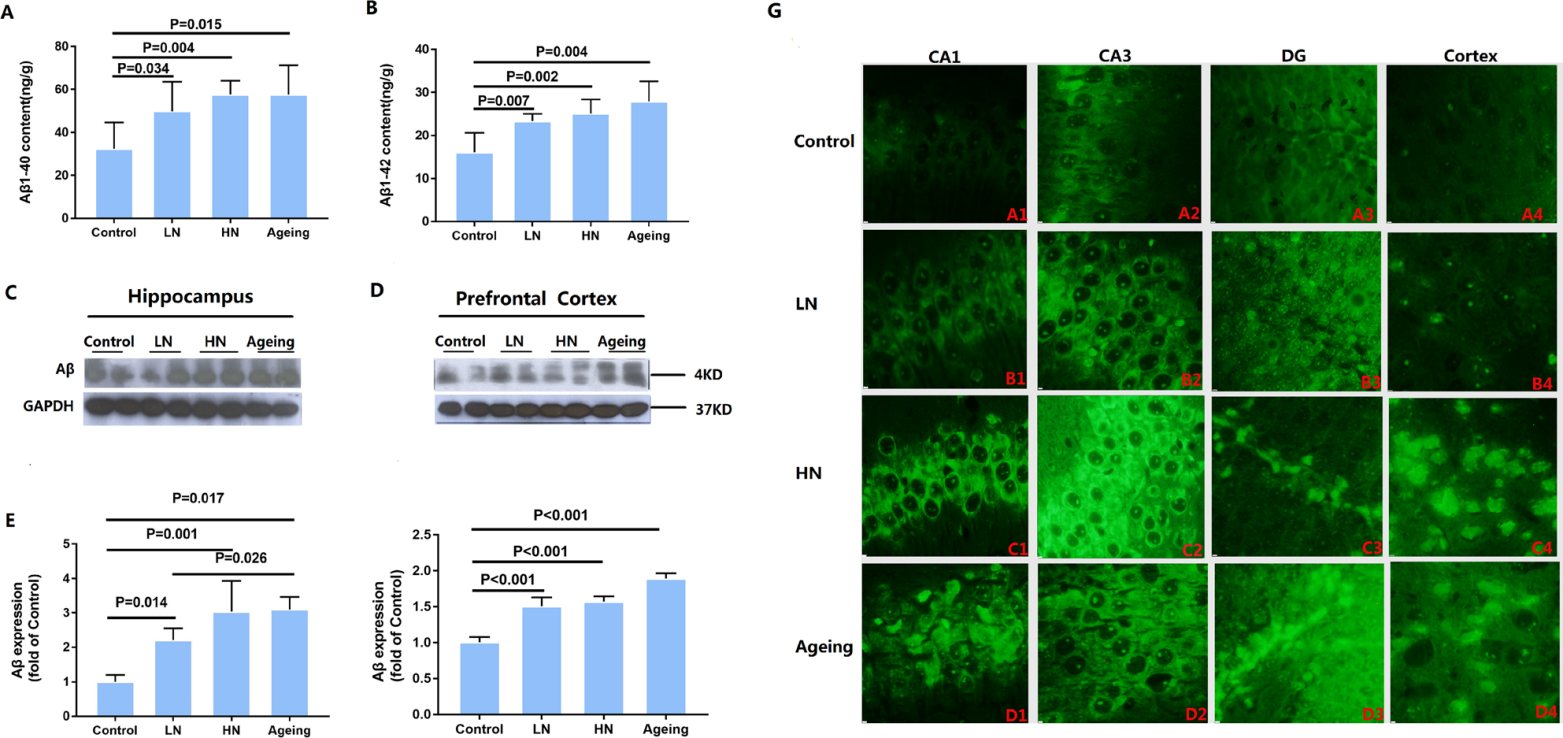
Chronic noise-induced changes of Aβ in SAMP8 mice. Quantification of Aβ 1–40 and Aβ 1–42 level by ELISA (**A**, **B**). Western Blot analysis of Aβ expression in the hippocampus (**C**) and PFC (**D**) in SAMP8 mice. Quantification of immunoreactive band density measured in Panels C and D, normalized against GAPDH. Quantification of immunoreactive band density measured in E and F. Data are represented as a percent change relative to the control (n = 6 per group). Data are shown as the mean ± standard deviation. HN, high-intensity noise exposure; LN, low-intensity noise exposure. Results were normalized as the control = 100%. The result of the distribution patterns of Aβ by thioflavin T (**G**). Representative images of hippocampal CA1 (A1, B1, C1, D1), CA3 (A2, B2, C2, D2), DG (A3, B3, C3, D3), and PFC (A4, B4, C4, D4) immediately after cessation of noise exposure. Scale bar = 15 μm. CA,Cornu Ammonis; DG, Dentate Gyrus; PFC, prefrontal cortex.

